# Phylogeography and historical demography of the Lusitanian snail *Elona quimperiana *reveal survival in unexpected separate glacial refugia

**DOI:** 10.1186/1471-2148-8-339

**Published:** 2008-12-19

**Authors:** Aude Vialatte, Annie Guiller, Alain Bellido, Luc Madec

**Affiliations:** 1 UMR CNRS ECOBIO, Université de Rennes 1, Campus de Beaulieu, 263 av. Gal Leclerc, CS 74205, 35042 Rennes Cedex, France

## Abstract

**Background:**

Present day distributions of Palearctic taxa in northern latitudes mainly result from populations having survived in local patches during the Late Pleistocene and/or from recolonizing populations from southern temperate refugia. If well-studied Mediterranean and eastern European refugia are widely accepted, some recent biogeographical assumptions still remain unclear, such as the occurrence of multiple glacial refugia in Iberia and cryptic refugia in northern Europe during the last glaciations. The Lusitanian snail *Elona quimperiana *has a remarkably disjunct distribution, limited to northwestern France (Brittany), northwestern Spain and the Basque Country. By describing the phylogeographical structure of this species across its entire range, the present study attempts to identify refugia and subsequent recolonization routes.

**Results:**

Results based on 16S and COI gene sequences showed that the low genetic diversity observed in the Brittany populations should be associated with a recent demographic expansion. By contrast, populations from Spain exhibit several differentiated lineages and are characterized by demographic equilibrium, while the Basque populations are the only ones harboring typical distinct haplotypes. The center of the star-like networks of both gene sequences is occupied by a common ancestral-like haplotype found in Brittany and Spain, which might have originated from the middle of Northern Spain (i.e. Asturias, eastern Lugo and western Cantabria). Estimates of the divergence time between the Spain-Brittany and Basque lineages strongly suggest that *E. quimperiana *survived the Pleistocene glaciations in distinct refugia on the Iberian Peninsula, one of which is situated in Picos de Europa, and the other in the Basque Country. The occurrence of a northern refugium in France cannot be rejected as of yet.

**Conclusion:**

Present results confirm the Iberian origin of the land snail *E. quimperian*a and strongly support the emerging phylogeographic hypothesis of multiple refugia in Iberia during the last glaciations. The scenario of a spatial expansion of *E. quimperiana *from an Iberian refuge located in Asturias to northern areas provides the most probable explanation for the present distribution of this land snail. By harboring distinct haplotypes, the Basque Country populations appear to be of great importance in terms of potential adaptation, long term persistence and hence, the conservation of *E. quimperiana*.

## Background

Geographically structured populations are the result of historical and/or contemporary demography. Some of them may experience either little or no genetic contact for long periods of time, due to physical, ecological or geographical barriers (e.g. [1-6]). More recent and rapid events, such as extinction, introduction or fragmentation, often linked to human activities, may also produce spatially partitioned populations (e.g. [7,8]).

For European species, the Quaternary glaciations are a major historical factor shaping the patterns of spatial population structure [9-13]. During these periods, the ice sheet induced the preservation of many species, but only in isolated refugia, resulting in population divergence via genetic drift and local selection [14]. The commonly recognized refugia in Western Europe during the Quaternary glaciations are on the Iberian and the Italian peninsulas [10,14,15]. It has been suggested that the northern regions of Europe were post-glacially colonized, generally from these refuges [10,14]. Recent phylogeographic and biogeographic studies have uncovered multiple refugia in Iberia (see [16] for a review). However, the number, location and habitat composition of these 'refugia in refugia' still remain poorly known. Scenarios other than the northern expansion from southern refugia have also been proposed. On one hand, cold-tolerant species, such as Mustela erminea [17], could have naturally expanded their repartition range during the Quaternary glaciations. On the other hand, the presence of additional putative refugia has been advanced to explain the distribution of poor cold-hardy species [18,19]. The hypothesis of local, but relatively recent and short-lived refugia much further north of the Mediterranean refugia is concordant with the discontinuity of the Pleistocene ice sheet in this region [20]. These temperate refugia are particularly recognized to explain the survival of some communities, such as freshwater fish [21]. The principal biome of unglaciated northern Europe during the late Pleistocene cold stages was the steppe tundra, *i.e*. a treeless vegetation type [22]. The presence of small isolated populations of trees in buffered local microclimates is also suspected in northwestern Europe during the Pleistocene; but, except for one controversial site in Belgium [18], no temperate forest refugia for animal species have actually been precisely localized in this part of Europe. Therefore, contemporary demographic factors are more likely to explain well-separated populations of poor cold-hardy species in Western Europe, such as human introductions (e.g. [23]), or the recent extinction of intermediate populations [24].

**Figure 1:**
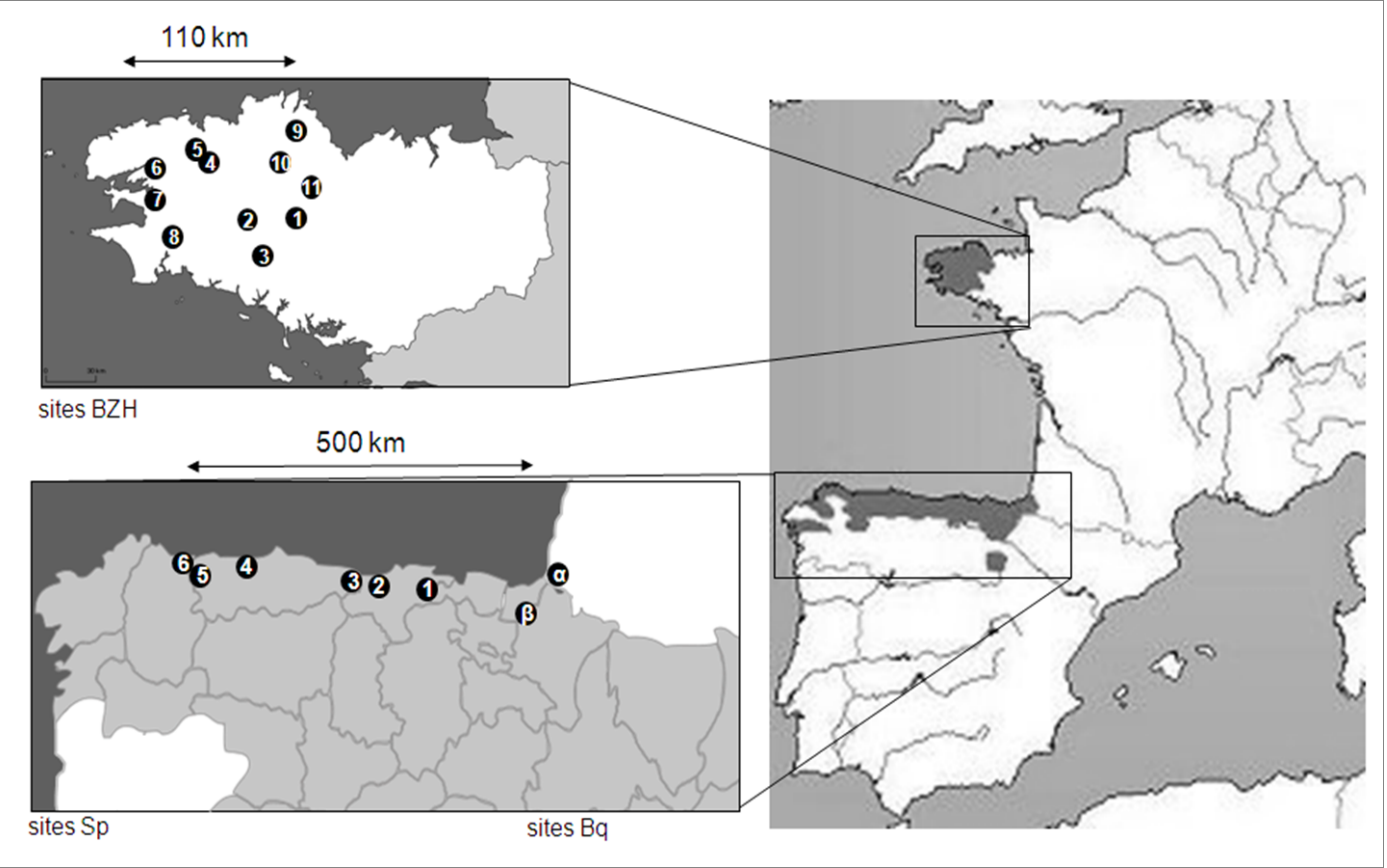
**Localities of the different populations sampled**. In (i) Brittany (France), numbered from 1 to 11, (ii) Spain, named from Sp1 to Sp8, and (iii) the Basque Country (France and Spain), called α and β. The samples cover the entire *E. quimperiana *distribution [25]. Modified from [75].

The land snail *Elona quimperiana *(Gastropoda, *Xanthonychidae *[25]) is a western Palearctic *Helicoidea *species with a highly disjunct distribution limited to Brittany in France (its name comes form the city of Quimper) and Northern Spain (Fig. 1, [26]). It is a so-called Lusitanian species, i.e. a species that typically has a disjunct distribution in Iberia and southwest Ireland, with either no or highly disjunct populations in the regions between both areas (i.e. England and France) [27,28]. *Elona quimperiana *therefore provides an excellent opportunity to shed more light on European historical biogeography. This species lives in temperate and humid deciduous forests, where it feeds on mycelia found on rotten, dead stumps (principally oak). Occasionally, it is coprophagous and necrophagous [29]. Like many other terrestrial gastropods, *E. quimperiana *has a relatively limited dispersal capacity and probably survived during the Quaternary glaciations through significant fluctuations in its distribution area, just as its deciduous forest habitat did [30]. The present study is based on phylogeographical analyses that are highly successful in discriminating past and present demographic factors that were likely to have influenced the distribution of populations. The aims are to (i) describe the phylogeographical structure of *E. quimperiana *throughout its entire range, and (ii) identify the refugia and the subsequent recolonization routes.

## Results

### Phylogenetic analysis

The length of 16S rDNA sequences ranged from 479 to 481 bp, with a total of 19 substitutions, including two indels at positions 123 and 420, while the length of the COI sequences was 683 bp, with a total of 47 substitutions. Eleven haplotypes were identified among the 54 snails analyzed for the 16S gene: eight from Spain, three from Brittany, and one from the Basque Country. Brittany had only one haplotype (haplotype 1) in common with Spain; this haplotype was the most frequent one, since 30 individuals shared it. Twenty-three haplotypes were obtained for the COI gene among the 81 individuals analyzed: eleven from Spain, six from Brittany (including one in common with Spain) and seven from the Basque Country. The most frequent haplotype (haplotype A), shared by more the half of the specimens, was also the only one common to both Brittany and Spain.

**Figure 2:**
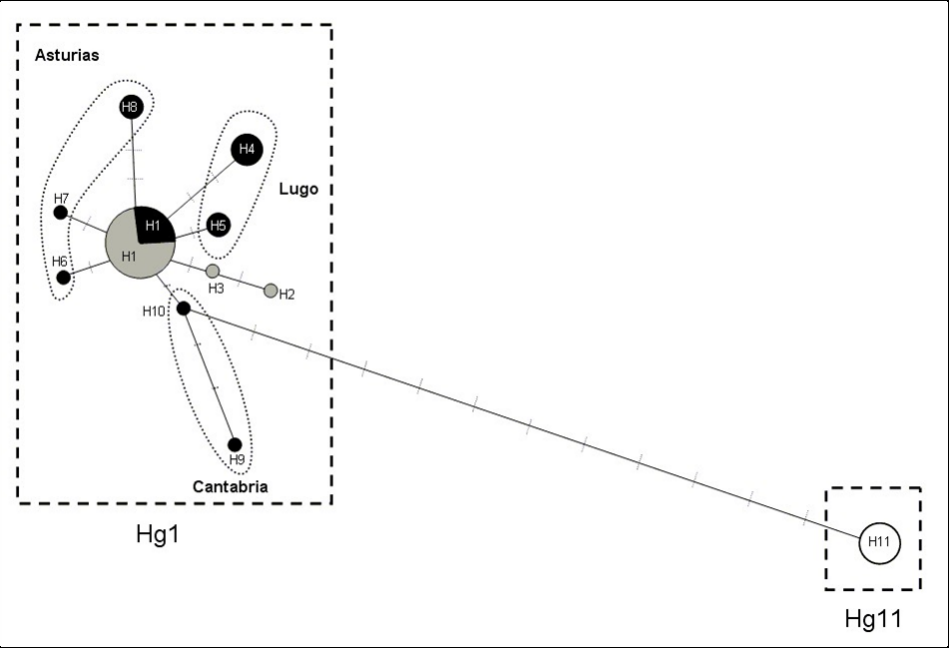
**Unrooted haplotype networks of *E. quimperiana *based on 16S rDNA**. Haplotypes originating from Brittany are represented in grey, those from Spain in black and those from the Basque Country in white. H1 to H11: names of the haplotypes; Hg1 and Hg11: names of the haplogroups. Haplotype H1 is present in Spain in the Asturias province, while the province origin of the other haplotypes are specified on the figure (i.e. Asturias, Cantabria and Lugo).

**Figure 3:**
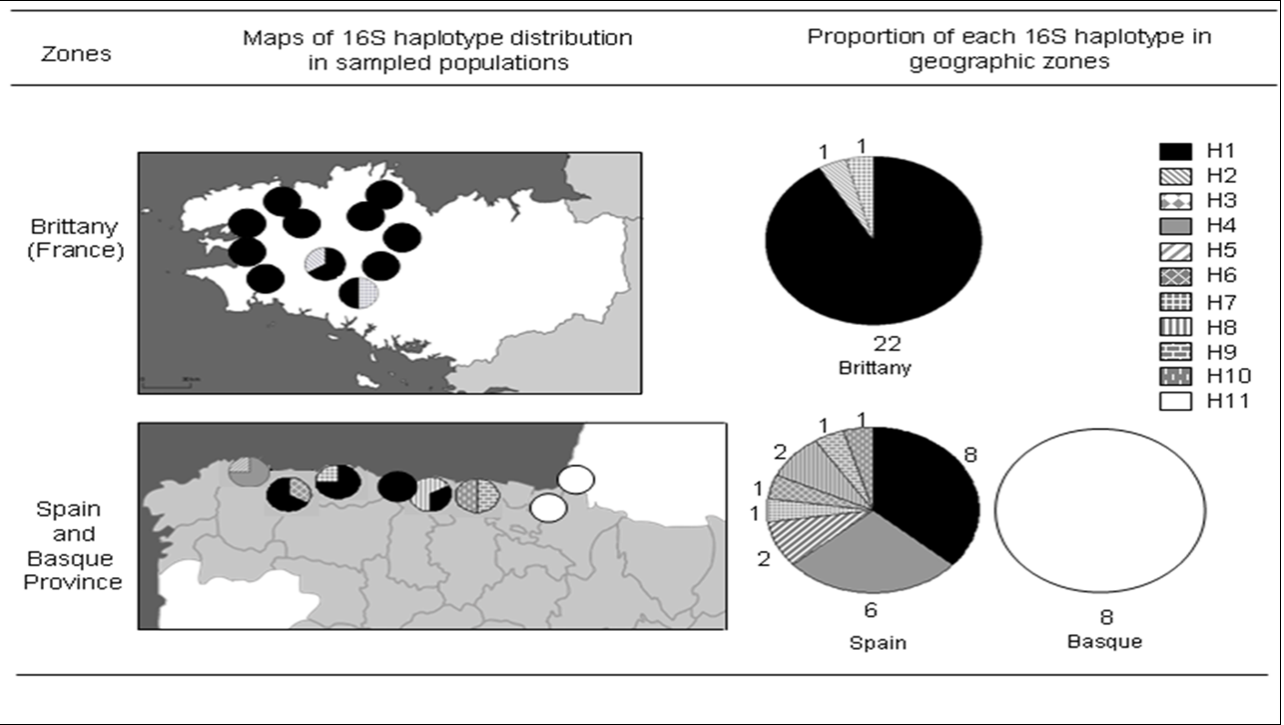
**Geographic distribution of *E. quimperiana *haplotypes in sampled populations across Brittany, Spain and the Basque Country for 16S rDNA**. Proportion of each haplotype in the different geographic zones is specified as the total number of individuals carrying these haplotypes.

The networks based on the 16S rDNA and COI sequences exhibited convergent results. The 16S network showed two haplogroups (Hg1 and Hg11; Fig 2). Hg1 comprised 10 haplotypes (46 individuals) that were scattered throughout Spain and Brittany. The most frequent haplotype in this group (H1; 30 individuals) is distributed throughout all of Brittany (except at BZH3 Pont Calleck) and occurs in the Spanish provinces of Lugo, Asturias and western Cantabria (sites Sp2, Sp3, Sp4 and Sp5; Fig 3). All of the other Hg1 haplotypes are represented by either a single or few specimens. Haplotypes 2 and 3 are specific to Brittany and were only found at BZH2 (Montagnes Noires) and BZH3 (Pont Calleck). In Spain, a total of seven private haplotypes were found in five out of six populations: haplotypes 4 and 5 were typical of Sp6 (Reme), haplotype 6 was specific to Sp5 (Gio), haplotype 7 was only found at Sp4 (Pravia), haplotype 8 was present in Sp2 (Cobreces), and haplotypes 9 and 10 were typical for the Cantabria province (site Sp1, Ramales de la Victoria).

The second haplogroup, Hg11, comprised only one haplotype (H11), which only occurred in Basque snails from Bqα (Sare) and Bqβ (Toloza Betulu).

**Figure 4:**
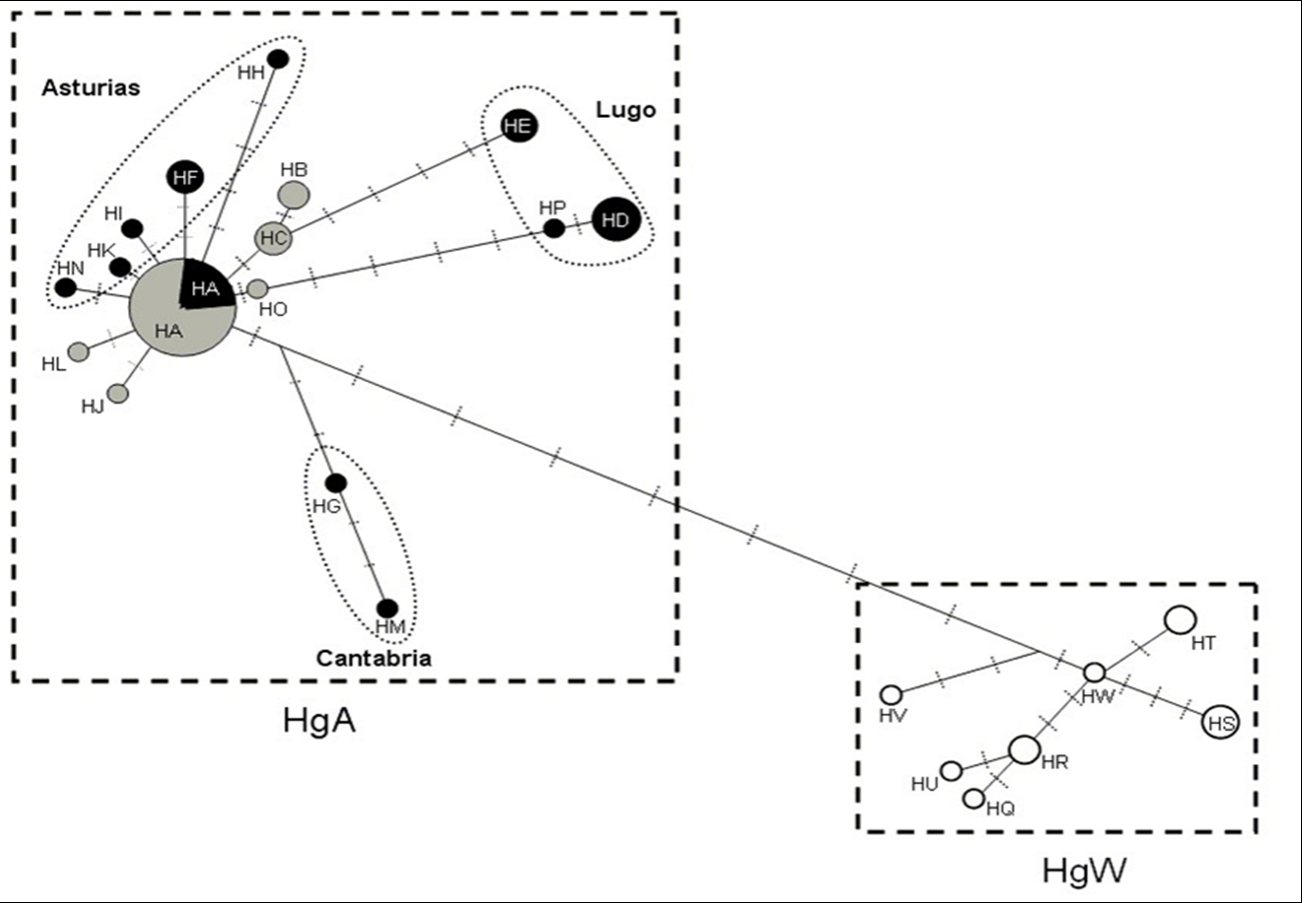
**Unrooted haplotype networks of *E. quimperiana *based on COI genes**. Haplotypes originating from Brittany are represented in grey, those from Spain in black and those from the Basque Country in white. HA to HW: names of the haplotypes; HgA and HgW: names of the haplogroups. Haplotype HA is present in Spain in the Asturias province, while the province origin of the other haplotypes are specified on the figure (i.e. Asturias, Cantabria and Lugo).

**Figure 5:**
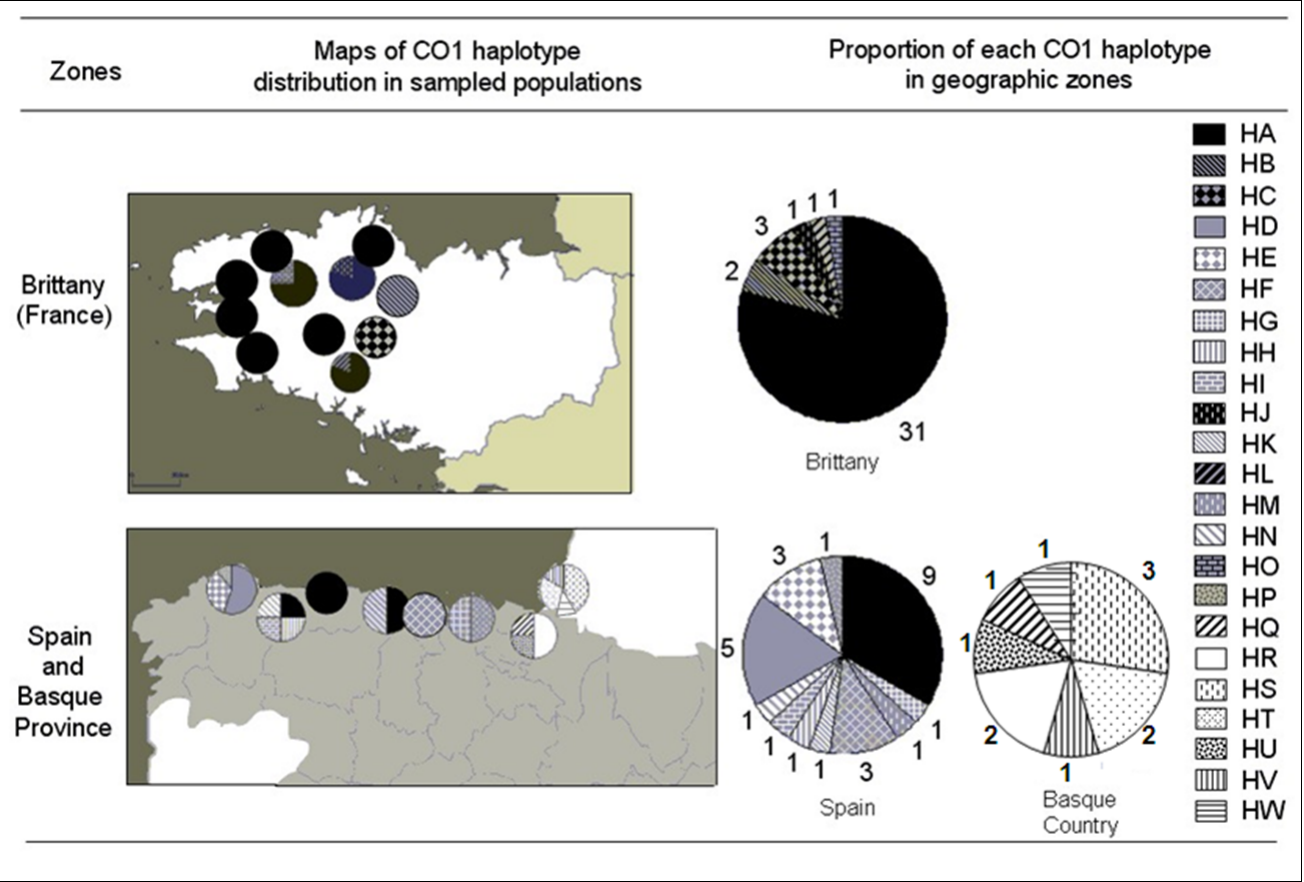
**Geographic distribution of *E. quimperiana *haplotypes in sampled populations across Brittany, Spain and the Basque Country for COI genes**. Proportion of each haplotype in the different geographic zones is specified as the total number of individuals carrying these haplotypes.

The populations of the two haplogroups formed by COI sequences (HgA and HgS) are the same as those found in Hg1 and Hg11 (Fig 4). HgA comprised 16 haplotypes (71 individuals), the most frequent of which was HA (44 individuals). The distribution of HA is comparable to that of H1, since it is found in both Brittany (except for the eastern sites, BZH1 and BZH11) and Spain (sites Sp3, Sp4 and Sp5). All of the other HgA haplotypes are represented by either a single or few specimens (Fig 5). The HB, HC, HJ, HL and HO haplotypes are specific to Brittany. Most of the 10 haplotypes specific to Spain are private. As for haplogroup Hg11, HgW exclusively comprised all of the Basque snails and included seven private haplotypes (HQ, HR, HS, HT, HU, HV and HW).

The genetic diversity estimates are given in Table 1. Obviously, both the haplotype and nucleotide diversities are much higher for both genes in the populations from Spain (^h^16S rDNA = 0.81 ± 0.01; ^π^16S rDNA = 1.89 ± 1.25; ^h^COI = 0.85 ± 0.05; ^π^COI = 4.73 ± 2.39), compared to those from Brittany (^h^16S rDNA = 0.16 ± 0.1, ^π^16S rDNA = 0.24 ± 0.32; ^h^COI = 0.34 ± 0.09; ^π^COI = 0.44 ± 0.4). While no variation is detected in the Basque populations for the 16S region, the gene and nucleotide diversities are relatively high for the COI gene (^h^COI = 0.91 ± 0.07; ^π^COI = 3.89 ± 2.11).

**Table 1:** Genetic polymorphism for different subsets of *E. quimperiana *populations according to their geographic origin.

*Population*	*N*_*haplotype*_		*N*_*ind*_		*h (SD)*		*π (SD)*	
	*16S*	*CO1*	*16S*	*CO1*	*16S*	*CO1*	*16S*	*CO1*
Brittany	3	6	24	43	0.16	0.34	0.24	0.44
					(0.10)	(0.09)	(0.32)	(0.40)
Spain	8	11	22	27	0.81	0.85	1.89	4.73
					(0.01)	(0.05)	(1.25)	(2.39)
Basque Country	1	7	8	11	0.00	0.91	0.00	3.89
					(0.00)	(0.07)	(0.00)	(2.11)
All	11	23	54	81	0.67	0.70	3.685.57	
					(0.07)	(0.06)	(2.21)	(2.70)

N_haplotypes_: number of haplotypes; N_ind_: number of individuals; h: haplotype diversity; π: nucleotide diversity; SD: standard deviation.

### Demographic analysis

**Figure 6:**
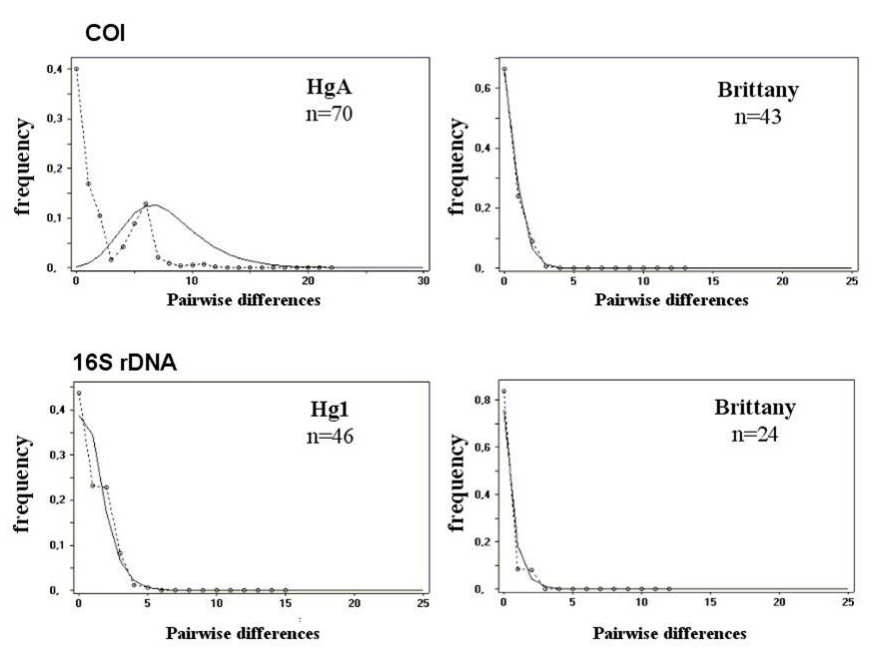
**Mismatch distribution of substitution differences between pairs of individuals from Brittany for 16S rDNA and COI genes**. The observed frequency is given as a dotted line. The expected distribution under a growth-decline model determined using the DNASP v3.5 program [54] is represented by a continuous line.

Hypotheses of demographic expansion were tested using Fu's *Fs *and Ramos-Onsins & Rozas' *R*^***2 ***^statistics (Table 2). Both tests yielded results that were consistent with a population expansion of both the Hg1 and HgA haplogroups, mainly driven by the significant expansion of the Brittany populations. The unimodal distribution of pairwise differences among the Brittany populations (Fig 6) and the non-significant raggedness index obtained from the mismatch distribution analyses (Table 2) are consistent with a model of recent expansion [31].

**Table 2:** Demographic analyses testing hypothesis of *E. quimperiana *population expansion.

*Statistics*	*All*		*Brittany*		*Spain*		*Brittany-Spain*		*Basque*	
	*16S*	*CO1*	*16S*	*CO1*	*16S*	*CO1*	*16S*	*CO1*	*16S*	*CO1*
Fu's FS	-3.89	-1.41	-3.90	-2.16	-0.8	-5.04	-5.96	0.00	-0.98	
FS *p-value*	0.56	0.13	0.03*	0.003***	0.11	0.39	0.002***	0.01**	N.A.	0.24
Ramos-Onsins & ozas'R^2^	0.06	0.11	0.06	0.09	0.09	0.05	0.04	N.A.	0.15	
R^2 ^*p-value*	0.32	0.10	0.14	0.05*	0.09	0.15	0.02*	0.02*	N.A.	0.28
Raggedness index r	0.11	0.05	0.57	0.21	0.05	0.05	0.12	0.08	N.A.	0.15
r *p-value*	1	0.76	0.73	0.65	0.49	0.48	1	1	N.A.	0.14

Analyses are using Fu's *Fs *and Ramos-Onsins & Rozas' *R*^2 ^statistics, for the whole sample (all) and four subsets of *E. quimperiana *populations (no sign: non-significant; *: *p-value *< 0.05; ***: *p-value *< 0.01).

### Bayesian estimation of time to the most recent common ancestor

The molecular-clock likelihood ratio-tests (LRT) showed no significant variation of the substitution rate among the branches of both the COI and 16S phylogenies. For 16S, 2ΔlogL = 24.5, df = 54, p = 0.99; for COI, 2ΔlogL = 29.5, df = 82, p = 0.99 with all codon positions, and 2ΔlogL = 66.33, d.f. = 82, p = 0.90, with the third-codon position only. Applying divergence rates ranging from 4 to 6.86% per Myr for the third codon of COI, the most recent common ancestor of all of the *E. quimperiana *populations (and 95% HPD) would have lived between 1 (0.64–1.45) to 0.6 (0.37–0.84) Myr ago (Table 3). The TMRCA, based on the COI-3rd base divergence rate, was estimated as being between 0.43 and 0.25 Myr for the Brittany-Spain lineage and between 0.31 and 0.18 Myr for the Basque lineage. Convergent time estimates were obtained for the Brittany-Spain clade with a 2.2% rate of evolution for COI (all bases; 0.29 Myr) and 2% for 16S (0.20 Myr). For the Basque clade, while a divergence rate of 2.2% for COI (all bases) leads to the same time value estimate (0.18 Myr), a four times lower divergence rate for 16S (around 0.5%/Myr) yielded an equivalent time estimate. In spite of homogeneous substitution rates among the branches in the 16S phylogeny, the molecular clock does not seem to tick regularly.

**Table 3:** Time to the most recent common ancestor (TMRCA; in million years, Myr) for all *E. quimperiana *sequences and for the two phylogenetic lineages defined.

*Gene*	*Standard molecular clock estimates*		*Divergence time estimates*		
			*All*	*Brittany-Spain*	*Basque Country*
**CO1**	**4% per Myr**	**mean (in Myr)**	**1.02**	**0.43**	**0.31**
	(third base)	*95% hpd upper*	*0.64*	*0.25*	*0.14*
		*95% hpd lower*	*1.45*	*0.65*	*0.52*
	**6.86% per Myr**	**mean (in Myr)**	**0.60**	**0.25**	**0.18**
	(third base)	*95% hpd upper*	*0.37*	*0.14*	*0.08*
		*95% hpd lower*	*0.84*	*0.38*	*0.30*
	**2% per Myr**	**mean (in Myr)**	**0.65**	**0.31**	**0.20**
	(three bases)	*95% hpd upper*	*0.42*	*0.17*	*0.09*
		*95% hpd lower*	*0.92*	*0.46*	*0.34*
	**2.2% per Myr**	**mean (in Myr)**	**0.59**	**0.29**	**0.18**
	(three bases)	*95% hpd upper*	*0.38*	*0.15*	*0.08*
		*95% hpd lower*	*0.84*	*0.42*	*0.31*
	**3% per Myr**	**mean (in Myr)**	**0.44**	**0.21**	**0.13**
	(three bases)	*95% hpd upper*	*0.28*	*0.11*	*0.06*
		*95% hpd lower*	*0.61*	*0.31*	*0.23*
**16S**	**0.5% per Myr**	**mean (in Myr)**	**2.39**	**0.81**	**0.17**
		*95% hpd upper*	*1.30*	*0.30*	*0.01*
		*95% hpd lower*	*3.70*	*1.36*	*0.41*
	**2% per Myr**	**mean (in Myr)**	**0.60**	**0.20**	**0.04**
		*95% hpd upper*	*0.32*	*0.08*	*0.002*
		*95% hpd lower*	*0.95*	*0.34*	*0.10*
	**10% per Myr**	**mean (in Myr)**	**0.12**	**0.04**	**0.01**
		*95% hpd upper*	*0.06*	*0.02*	*0.0004*
		*95% hpd lower*	*0.19*	*0.07*	*0.02*

Results of Bayesian analyses are based on 16S rRNA and CO1 (considering the three bases or the third-codon position only) variation.

## Discussion

In spite of its name, an ancient Iberian origin of *Elona quimperiana *is obvious, as has been suggested earlier [26]. This is strongly supported by the observation that most of the mtDNA diversity is found in Spain, and by the fact that Spanish haplotypes have a central position in both of the star-like sequence networks. Our genetic comparison of populations from the two disjunct distribution areas of *E. quimperiana *reveals that the genetic differentiation does not mirror the large geographical discontinuity of the species range.

**Figure 7:**
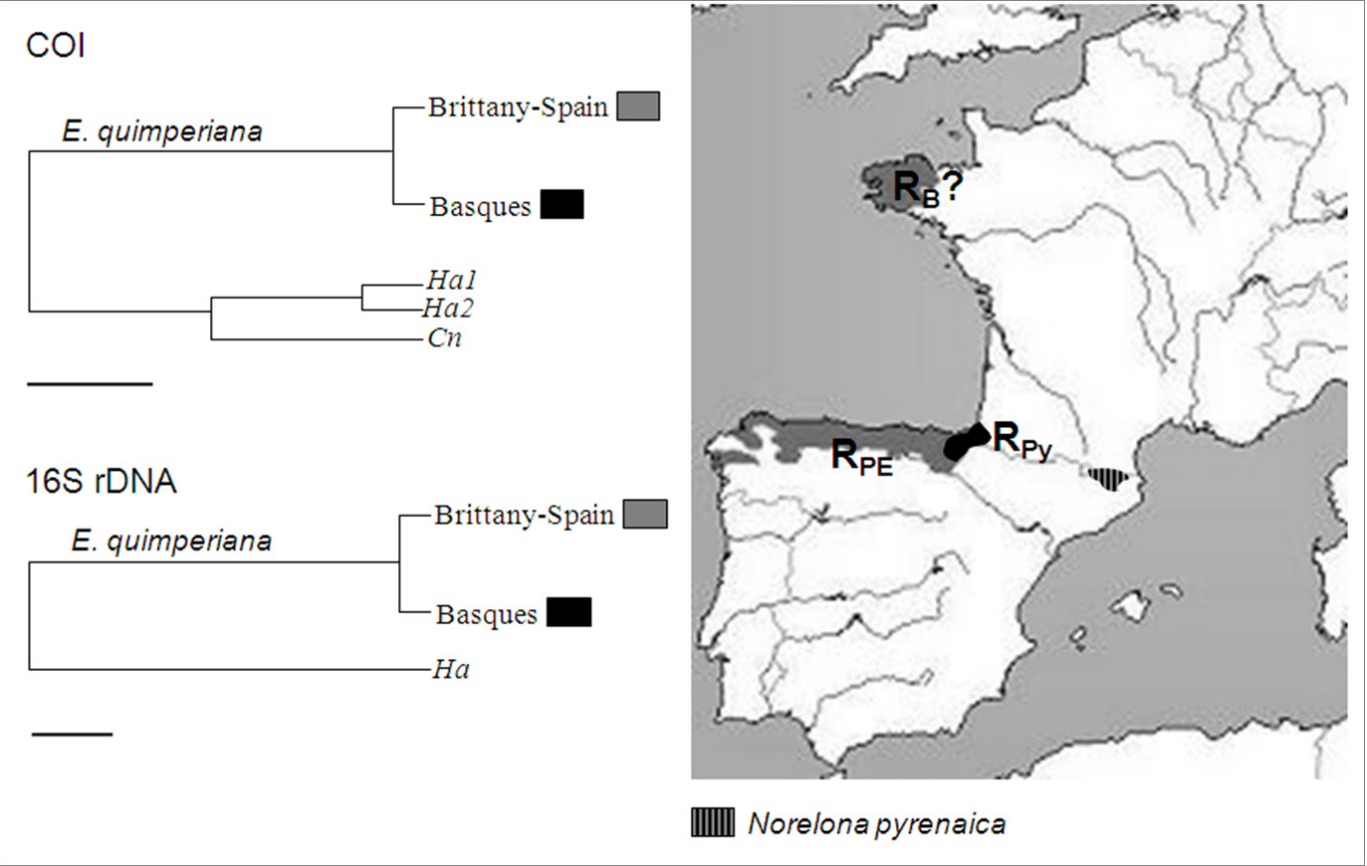
**Synthetic representation of the phylogenetic structure of *E. quimperiana *populations from Brittany, Spain and the Basque Country**. Ha and Cn: outgroups corresponding respectively to *Cantareus aspersus *and *Cepaea nemoralis*. The scaled bar under the trees represents a 0.1% sequence divergence. Genetically sustained refugia during the glaciations are localized in Picos de Europa in Spain (R_PE_) and the Basque Country in the Pyrenees (R_PY_), while a putative third refugia during the last glaciations may be hypothesized in Brittany (R_B_?). The localized distribution of the related species *Norelona pyrenaica *is specified (eastern Pyrenees).

Actually, the genetic divergences isolate the Basque populations from a homogeneous genetic group comprising the Spanish and Brittany populations. Both the 16S rDNA and COI genes show specific Basque haplotypes, forming separate clades in the two haplotype networks. The low level of genetic diversity, especially for the 16S gene, the occurrence of private haplotypes, and the peripheral location suggest that the Basque populations may form a parapatric race [14], although no morphological peculiarities could be detected. Such peripheral populations frequently mark a threshold of environmental variation, beyond which the species cannot expand [32]. As the split between the Basque and central Spanish populations would have occurred between 1 Myr to 600,000 BP, it can be most easily interpreted by assuming that the species survived the Pleistocene Ice Ages in separate glacial refugia on the Iberian Peninsula [16]. Indeed, the occurrence of 'refugia within an Iberian refugium' is now largely supported by data from a range of organisms. Among the seven putative terrestrial refugia identified in this area, those located along the Picos de Europa (north of Spain, [33-37]) and in/or near the Pyrenees [34,36,38] coincide quite strikingly with the phylogeographic structure we obtained (Fig 7). Our results thus support the spatial subdivision hypothesis of an Iberian Peninsula glacial refugia [16], and help to refine the Pyrenees refugium theory by suggesting the Basque Country as a refuge zone during the Pleistocene glaciations. Moreover, the presence of the closely related species *Norelona pyrenaica*, which is endemic in the eastern Pyrenees, where *E. quimperiana *does not live (Fig 7, [39]), suggests the occurrence of another separate refuge in this part of the Pyrenees, which is probably influenced by Mediterranean climates. Contemporary anthropogenic effects may also influence the population structure of *E. quimperiana *since estimates of the split of the Basque populations match glacial and postglacial times (Table 3). Recurrent bottlenecks, due to habitat fragmentation and the alteration of natural forest habitat in the Basque Country [40], could have accentuated this genetic divergence (e.g. [41,42]). This could explain the discrepancies in the divergence time estimates between the two genetic markers, while a strong concordance has been observed in the divergence time estimates for other *E. quimperiana *lineages. By harboring distinct haplotypes, the Basque Country populations appear to be of great importance in terms of potential adaptation, long term persistence and hence, the conservation of *E. quimperiana*.

Besides the strong genetic divergence of the Basque lineage, the present results also show that in the Spain-Brittany lineage, (i) the genetic diversity is higher in Spain, (ii) the Spanish and Brittany populations share one common haplotype for each gene, which is, moreover, the most frequent in the two areas, and (iii) the Spanish populations are at demographic equilibrium, while the Brittany ones would have experienced a recent expansion. Three scenarios might be considered in order to explain the origin of Brittany populations: 1 – a recent introduction by human activities, 2 – an ancient northward colonization preceding the last glacial period, as demonstrated for other Lusitanian species [19], 3 – a spatial expansion after the Last Glacial Maximum (LGM), also from native Spanish populations [24]. In the first case, it is expected to have haplotypes in common with the source and introduced zones, as illustrated by 16S rDNA-H1 and COI-HA. All of the other haplotypes found in Brittany are specific to this area, suggesting that they probably appeared in Brittany after an introduction. However, such post-introduction genetic diversification is more typical for species with high colonization capabilities, which is not the case for *E. quimperiana *[29]. Moreover, related life history features, inconsistent with human activities or production, render multiple introductions improbable. All of these points make the first assumption rather unlikely.

The second hypothesis conforms to predictions of the effects of the ice ages on genetic variability in populations: populations with historical distributions in northern latitudes present lower genetic diversity, with fewer differentiated lineages, than southern populations (Hewitt [14] and Avise [9]). This has been demonstrated in other ectotherm species [43]. An ancient expansion of populations from Iberia through France during the Pleistocene interglacial periods may be plausible, since during these periods, the environmental conditions in Western Europe (e.g. high precipitation and cool temperatures) were particularly favorable to *E. quimperiana*, all the more so given that oak was a dominant tree species [30]. Under this hypothesis, a subset of southern haplotypes (from the Picos de Europa refugium) would have expanded northward, while others (from the Pyrenees refugium) remained in the south. Glaciations would have subsequently eradicated most of the *E. quimperiana *populations, except in the Iberian refugia and in a northern refuge zone. Indeed, small microenvironmentally favorable zones might have been created by oak trees in Western Europe [44]. According to paleoclimatic reconstructions, the local environmental conditions in close proximity to the arboreal vegetation were indeed warmer and more humid than regional simulations of the full-glacial climate [45]. Within close proximity to the trees, poor cold-hardy flora and fauna were also able to survive the extremes of the LGM. It is also possible that the Pleistocene refuge in northwestern France may involve caves, as suspected in Belgium for other poor cold-hardy animals and deciduous trees [18]. *Elona quimperiana *is indeed frequently observed in caves [46], where it survives by coprophagous and necrophagous feeding.

Unfortunately, our results cannot distinguish between the second and the third scenario. The latter implies a colonization of Brittany after the last glaciations. As illustrated by oak (*Quercus sp*.) dispersal in Great Britain some 10 000-9500 years ago [47], the oceanic climate of the west coast in France could have allowed a rapid spread of *E. quimperiana *from southern refugia to northern areas. Since this snail is strongly associated with oak forests, its dispersal may follow the tree expansion pattern. Human deforestation in intermediate areas between the Basque Country and Brittany, around 1000-500 years ago, may have created induced open habitats that are hostile for *E. quimperiana *[48], thus potentially explaining its present disjunct distribution.

There is no fossil record of *E. Quimperiana *due to the extreme fragility of its thin shell. Although the occurrence of a northern refugium should not be discarded *a priori*, an exhaustive sampling of the southern distribution range (based on a new imperative inventory, since many recorded sites appear to be presently disturbed by human activities) where Pleistocene refugia are obvious, appears necessary in order to clarify the colonization history of Brittany by *E. quimperiana*.

## Conclusion

The present results confirm the Iberian origin of the land snail *E. quimperian*a and reveal a genetic differentiation separating a Basque lineage from monophyletic clade grouping sequences from Brittany and Spain. Divergence time estimates suggest the coexistence of these lineages in the Iberian refugia during the LGM, and strongly support the hypothesis of 'refugia in refugia' during glaciations. The identification of Iberian lineages allows the precision of the glacial refugia in the Basque Country and Asturias locations. Moreover, the species' ecology (inhabiting deciduous oak forests) allows researchers to sharpen their focus on the type of habitat present in these refugia during the glaciations. The scenario of a spatial expansion *of E. quimperiana *from the Iberian refugia located in Asturias to northern areas in France is the most probable for explaining the present distribution of the land snail. Inferences based on a spatial genetic structure analysis should be helpful to discriminate between a pre-glacial expansion implying the existence of glacial refugia in Brittany, or a post-glacial expansion followed by the human oak deforestation along the Atlantic coast in France.

## Methods

### Sample collection

Tissue samples were taken from a total of 81 individuals legally collected in 19 field populations across Brittany (France, sites BZH1 to BZH11), the Basque Country (France and Spain, sites BQα and BQβ) and Northern Spain (sites Sp1 to Sp6) between 2006 and 2007 (Fig 1). The other sites where this species was previously inventoried (see [26] for a review) were thoroughly searched, but no snails were found. The principal threat to this species is probably deforestation in those areas.

### DNA Extraction, Amplification and Sequencing

Foot tissue was dissected from each individual and placed in a 10% chelex solution, with 15 μl of proteinase K (10 mg/ml). Samples were incubated overnight at 55°C, and then briefly vortexed, before being boiled for 2 × 15 min. After a centrifugation at 10,000 g for 5 s, the DNA in the supernatant was used in the subsequent amplifications.

DNA was amplified by the polymerase chain reaction (PCR) for the mitochondrial 16S rDNA on a set of 54 individuals, and mitochondrial cytochrome *c *oxydase-1 (COI) on 81 individuals. The 16S rDNA and COI gene fragments correspond, respectively, to a 480 base pair sequence using the primers 984 (5'-CGCCTGTTTAACAAAAACAT-3') and 16S2 (5'-CTGGCTTACGCCGGTCTG-3') [49], and to a 683 base pair sequence using the primers FCOI (5'-ACTCAACGAATCATAAAGATATTGG-3') and RCOI (5'-TATACTTCAGGATGACCAAAAAATCA-3') [50,51]. PCRs were carried out in 25 μl volumes containing 0.5 μl (10 μM) of each forward and reverse primers, 12.5 μl of the Diamond DNA Polymerase – 500 (Bioline^®^), 10.25 μl of UP water and 1.25 μl of DNA template. The PCR conditions for the 16S rDNA gene were an initial denaturation step of 94°C (3 min), followed by 35 cycles of 94°C (30 s), 49°C (30 s), 72°C (40 s) and a final extension phase at 72°C for 4 min. Those for the COI gene were an initial denaturation step of 94°C (5 min), followed 35 cycles of 94°C (45 s), 52°C (45 s),72°C (1 min) and a final extension phase at 72°C for 7 min. The PCR products were sequenced in both directions on an automated sequencer using the PCR primers (PE Applied Biosystems 310 Genetic Analyser, UMR 6553; plate-forme de séquençage génotypage OUEST-genopole^®^).

### Sequence analysis

Sequences were aligned using CLUSTAL W [52] and were manually checked in the BIOEDIT sequence editing program [53]. The sequences have been submitted to GenBank (Accession N° FJ491809-FJ491943). Sequence polymorphism analyses (haplotype diversity h and nucleotide diversity π) were carried out with DNASP version 4.10. [54] and ARLEQUIN version 3.1 [55]. The land snails *Cantareus aspersus *and *Cepaea nemoralis *were used as outgroups.

### Phylogenetic analysis

Phylogenetic relationships among haplotypes were estimated using maximum likelihood (ML) and Markov-Chain Monte-Carlo (MCMC) bayesian-based inference (BI) methods. ML and BI analyses were performed using PAUP* version 4.0b10 [56] and MRBAYES v. 3.1.1-p1 [57] softwares, respectively. The best fit model of nucleotide substitutions was selected prior to ML and BI using the Akaike Information Criterion [58]. The software MrAIC v.1.4.2 [59] was used to evaluate the fit of the data to 24 different models of nucleotide substitutions. The resulting best fit model was the *F81 *model [60] for the 16S rDNA region, with an unequal rate for base frequencies and equal rates for transitions and transversion. For the COI gene, the best fit model was the *HKY *model [61] with two different rates for transitions and transversion, unequal base frequencies, a parameter for invariable sites (*I*) and a gamma distribution parameter that describes the rate variation across variable sites (*Γ*). The model of nucleotide substitution was incorporated in PHYML[62] and in MRBAYES for the ML and BI analyses respectively. For the ML analysis, the robustness of inferences was assessed by bootstrap resampling using 1,000 repetitions. For the Bayesian analysis, the posterior probabilities of trees and parameters were approximated with Markov-Chain Monte-Carlo and Metropolis coupling. We ran two independent MCMC analyses with four chains and a temperature set to 0.2. Each chain was run for 2,000,000 cycles with trees sampled every 100 generations. Posterior probabilities were obtained from the 50% majority rule consensus of trees sampled after discarding the trees saved before chains reached apparent stationarity (i.e. a 'burn-in period" of 50,000 generations for 16S rDNA).

We used network-based approaches for intraspecific phylogenetic analyses to deal with the low divergence among individuals and the possible persistence of ancestral nodes [63]. The median joining algorithm implemented in the NETWORK v. 4.2.0.1 software [64] was used with the default settings for constructing networks (weight = 10 and **e **= 0), and we simplified the median networks that contained all of the possible equally shortest trees by running the MP (maximum parsimony) calculation option.

### Demographic analyses

The demographic history of the populations was inferred using two of the most powerful tests of population expansion: (i) Fu's *Fs *[65], which represents the probability of observing a similar or a higher number of haplotypes in a random neutral population given the observed value of theta. In populations that have experienced recent expansion, large negative values of *Fs *are expected, due to an excess of rare alleles; (ii) Ramos-Onsins & Rozas' [66]*R*^***2 ***^statistics, representing the difference between the number of singleton mutations and the average number of pairwise differences. Recent population expansions are expected to be associated with low values of this parameter. *R*^2 ^and *Fs *statistics were estimated using DNASP v. 4.10.9 [54] and ARLEQUIN v. 3.1 [55] respectively, and their significance was assessed using 1,000 coalescent simulated resamplings. In order to evaluate the possible historical events of population growth or decline, mismatch distribution analyses were also performed [67]; in the first case, populations show unimodal distributions, while in the second case the populations at demographic equilibrium present multimodal distributions [68]. Mismatch distributions were computed for each haplogroup and compared to the expected distributions obtained under a model of sudden expansion.

### Molecular clock and divergence times

A molecular-clock likelihood ratio-test (LRT, [47,50]) was first conducted on the 16S rDNA and COI sequences (considering the three bases and only the third base of codon) to ascertain whether or not the *Elona *sequences were evolving at a homogeneous rate along all of the branches in the phylogenies. This test compares the log-likelihood of the ML trees under alternative molecular clock assumptions (i.e. a relaxed vs. an enforced molecular clock). The statistic **A **= 2(lnL1–lnL2) could be compared with a distribution with (*n*-2) degrees of freedom (where *n *is the number of sequences). Divergence dates were computed using BEAST[69] in order to provide estimates of time to the most common ancestor (TMCRA) for *E. quimperiana *populations, along with the TMCRAs of the Brittany-Spain (Hg1 and HgA) and the Basque lineages (see Results). An HKY model of nucleotide substitution was employed with a sequence divergence rate based on several different published rates for molluscs. While the divergence rates for 16S rDNA are estimated at around 2% per Myr for animals [70], the estimates for gastropods vary between 0.5 – 0.6% [71,72] up to an accelerated rate of 10% per Myr, as seen in the land snail *Mandarina sp *[73]. For COIGENE, we used rates from bivalve species inferred from geological events [74]. These rates range from 0.03 to 6.84% per Myr, depending on the codon position considered. The number of mutations recorded for the COI first- and second-positions (7 of 456 nucleotides) and COI third-positions (39 of 227 nucleotides) may reflect a variation in rates of substitution among these types of sites, in favor of a higher rate for COI third-positions. Given that the clades of the BI tree, based on the two first codon positions of COI, appeared to not be statistically supported (result not shown), we applied COI third-positions rates only when estimating divergence times (4 to 6.86% per Myr). These time estimates were then used to infer the 16S rDNA mutation rates by testing several substitution rates (0.5%, 2% and 10% per Myr).

## Authors' contributions

AV and LM conceived and designed the research; AV, AB and LM conducted the samplings; AV performed the DNA extractions, AG and AV analyzed the data, and AV and AG wrote the paper.
